# Stimulation intensities influence the effects of spinal cord stimulation in disorders of consciousness: an fNIRS study

**DOI:** 10.1117/1.NPh.12.3.035003

**Published:** 2025-08-07

**Authors:** Aoxuan Liu, Qianqian Ge, Liqin Jiao, Hao Peng, Yuhang Sun, Shuai Han, Qin Zhang, Juanning Si, Jianghong He

**Affiliations:** a Beijing Tiantan Hospital, Capital Medical University, Department of Neurosurgery, Beijing, China; b Capital Medical University, Beijing, China; c Beijing Fengtai Rehabilitation Hospital (Tieying Hospital), Beijing, China; d Beijing Information Science and Technology University, School of Instrumentation Science and Opto-Electronics Engineering, Beijing, China

**Keywords:** functional near-infrared spectroscopy, disorders of consciousness, spinal cord stimulation, intensity

## Abstract

**Significance:**

Disorders of consciousness (DOCs) pose significant challenges for therapeutic intervention. Spinal cord stimulation (SCS) has emerged as a promising neuromodulation technique for treating DOC patients. However, the selection of optimal SCS stimulation parameters, particularly intensity, lacks objective standards, and considerable variations in the configuration of stimulation intensity are evident among different research groups in previous studies.

**Aim:**

We aim to systematically evaluate the effects of different stimulation intensities of SCS using functional near-infrared spectroscopy (fNIRS) to further optimize the efficacy of SCS.

**Approach:**

Eleven DOC patients with implanted SCS devices were recruited. Four different stimulation intensities based on individual motor thresholds were used: low (50%), threshold (100%), medium (125%), and high (150%). Hemodynamic responses were recorded using fNIRS, and the mean, peak, and net area under the curve values of hemodynamics, as well as the activated channel count, were analyzed, mainly focusing on two regions of interest: the prefrontal cortex (PFC) and the temporo-parietal junction (TPJ).

**Results:**

An inverted U-shaped dose–response curve was observed. The medium-intensity group triggered the most significant hemodynamic responses. The high-intensity group evoked less pronounced responses and showed negative responses post-stimulation. The threshold-intensity group exhibited positive responses but less pronounced than the medium- and high-intensity groups. Conversely, the low-intensity SCS evoked a decreased response. The medium-intensity SCS also resulted in the highest number of activated channels and maintained the highest total hemoglobin concentration level during the inter-stimulus interval. Differences in brain region responses to SCS intensity were observed, with the PFC tolerating higher intensities and the TPJ having a narrower therapeutic window.

**Conclusions:**

Our findings illustrate that the medium-intensity SCS provides the optimal hemodynamic effect. The observed inverted U-shaped dose–response curve underscores the importance of precise parameter adjustments in SCS for DOC patients to maximize efficacy and to avoid overstimulation or insufficient activation.

## 1 Introduction

Disorders of consciousness (DOCs) encompass a spectrum of conditions characterized by varying degrees of impaired awareness and responsiveness, mainly including coma, unresponsive wakefulness syndrome [UWS also known as vegetative state (VS)], and minimally conscious state (MCS).^1^ DOCs often result from severe brain injuries, such as traumatic brain injury (TBI), stroke, and intracerebral hemorrhage (ICH). Although some DOC patients may regain consciousness, the prognosis for DOC patients is generally poor, especially when the condition has progressed to chronic DOC,^2^ with limited therapeutic options available to facilitate recovery.^3^^,^^4^ Evidence indicates that DOC patients exhibit significantly reduced cerebral blood flow in consciousness-related regions including prefrontal cortex (PFC), thalamus, and temporo-parietal areas, and this hypoperfusion correlates with poorer prognosis.^5^ Spinal cord stimulation (SCS) is a promising neuromodulation technique that can improve this hypoperfusion.^6^^,^^7^ There is increasing evidence that SCS can improve the level of consciousness in patients with DOC, as measured by the Coma Recovery Scale-Revised (CRS-R) score.^8^^–^^10^ A recent review reported that SCS has an effectiveness rate of 42.3% for patients in a VS and 69.0% for those in an MCS.^11^

As the efficacy of SCS in DOC is fundamentally mediated through its ability to enhance cortical blood flow, with stimulation parameters critically determining the magnitude and spatial distribution of this hemodynamic response, our previous research indicated that specific frequencies and inter-stimulus intervals (ISIs) can significantly improve cortical blood flow in DOC patients.^12^^,^^13^ Optimal parameter settings are crucial for maximizing benefits while minimizing potential side effects. This study aims to investigate how different SCS intensities affect the efficacy of SCS in DOC patients. Emerging evidence suggests that neuromodulation therapies often exhibit non-linear intensity–response relationships. Studies across various neuromodulations—including transcranial magnetic stimulation (TMS)^14^ and transcranial direct current stimulation (tDCS)^15^^–^^17^—have consistently demonstrated inverted U-shaped dose–response curves, where moderate intensities produce optimal effects while both lower and higher intensities show reduced efficacy. We hypothesize that SCS may follow a similar pattern, with medium intensities producing more balanced cortical activation than either low or high intensities. However, in practice, due to the unique characteristics of patients with DOC—such as impaired cognitive, motor, and language functions—they are unable to provide feedback that can guide adjustments. In most cases, only motor neuron activation, upper limb twitches, can be observed, making it difficult to assess the actual effects. In previous studies, the selection of SCS intensities has varied widely, with no systematic research conducted to identify the optimal intensity. Some studies have determined stimulation intensity based on motor responses, such as the intensity that induces upper limb twitches^18^ or slightly above/below that level,^7^^,^^19^ without providing specific justification. Other studies have used a fixed absolute intensity, such as 3 V.^20^^,^^21^ However, as motor thresholds vary among different patients, the intensity required to activate neural pathways differs across individuals, making it inappropriate to apply a fixed intensity to all patients. These variations in stimulation intensity likely contribute to the inconsistent results observed across different studies and may also influence the therapeutic effects of SCS in the field of DOC. Therefore, the development of objective and validated stimulation protocols that account for patient-specific variability is crucial for optimizing SCS therapy.

In clinical practice, given the inability to obtain direct feedback from patients with DOC, evaluating treatment effectiveness is particularly challenging and necessitates the use of neuroimaging techniques.^22^^,^^23^ Functional magnetic resonance imaging (fMRI) is widely used in the field of DOC^24^ and has shown improvements in brain metabolism following treatment such as transcranial magnetic stimulation.^25^ However, fMRI presents limitations when evaluating the effects of electrical stimulation during the period when the stimulation is “on,” primarily due to the interference from the stimulation itself and associated safety concerns. In addition, fMRI is expensive, cumbersome, and has strict subject constraints, making longitudinal bedside repeated measurements impractical. Electroencephalography (EEG) has also been used to assess the effects of SCS on DOC patients.^20^ However, as an electrical signal-based technique, its results are less interpretable. There is a study suggesting that even patients who ultimately show clinical improvements may not exhibit detectable changes in EEG after a single 15-min SCS session.^19^ Our previous studies have demonstrated that SCS can induce clear and immediate changes in cerebral blood flow that can be directly measured by functional near-infrared spectroscopy (fNIRS) during the stimulation session.^12^^,^^13^ In recent years, fNIRS has become increasingly popular in the assessment of DOC due to its portability and real-time monitoring capabilities.^26^^,^^27^ Unlike fMRI, fNIRS is optically imaging and provides better temporal resolution, making it ideal for studying dynamic brain processes when the stimulation is on.^28^ FNIRS also surpasses EEG in spatial resolution and can measure cortical hemodynamics and metabolic activity.^29^ These features position fNIRS as an ideal tool for monitoring cerebral blood flow and assessing the effects of different parameters of neuromodulation therapies, such as SCS in DOC patients.

In this study, we employed a high-density fNIRS device with 106 channels to comprehensively monitor cerebral hemodynamics in DOC patients. The high-density coverage provided by this device allows for detailed analysis of different brain regions, including the PFC and temporo-parietal junction (TPJ). The PFC, located in the anterior part of the brain, is a region critically involved in conscious processing and higher-order cognitive functions.^30^^,^^31^ In DOC patients, enhancing PFC activity via SCS may improve cognitive responses and awareness. Previous studies have shown a significant increase in functional connectivity in the PFC following SCS.^9^ However, there is currently a debate on whether consciousness recovery is primarily supported by frontal (e.g., PFC) or posterior (e.g., TPJ) networks and structures.^32^ Notably, TMS studies have been conducted to explore this functional dissociation.^33^ Consistent with this line of inquiry, we also included the TPJ in our analysis. Located in the posterior part of the brain, the TPJ is implicated in multisensory integration, social cognition, and attentional processes.^34^ Modulating TPJ activity through SCS could enhance sensory processing and potentially improve patients’ interaction with their surroundings.

Stimulation intensity plays a critical role in determining the therapeutic efficacy of SCS, yet previous studies have not systematically established an optimal intensity. Instead, researchers have used various, often inconsistent, criteria to define the stimulation intensity, leading to variability in outcomes. To address this gap, in this study, fNIRS technology was utilized to investigate the cerebral hemodynamic changes elicited by different SCS intensities, with a particular focus on how these effects vary across different brain regions. By identifying the intensity that produces the most effective neural activation, we aim to develop a standardized approach for determining optimal stimulation intensity. This could help unify the protocols used by different centers and ultimately contribute to better management and enhance the therapeutic effectiveness of SCS in patients with DOC.

## 2 Materials and Methods

### 2.1 Participants

Eleven subjects (eight males and three females, ages 18 to 62 years) from the Department of Neurosurgery, Beijing Tiantan Hospital and Beijing Fengtai Rehabilitation Hospital (Tieying Hospital), were recruited. Each patient had implanted an SCS device (SCL302B, Rishena Medical Ltd., Changzhou, China) for at least 2 weeks, but the stimulation had not been activated. In this study, the inclusion criteria for the subjects were as follows: (1) ages between 18 and 65, (2) diagnosed with UWS or MCS using the CRS-R, (3) with etiology including either TBI or ICH, (4) a documented DOC duration >28 days from official diagnosis, and (5) consent provided by caregivers. The exclusion criteria included the following: (1) a history of epilepsy, neurological disorders, or major psychiatric disorders; (2) long-term use of sedative drugs; and (3) severe uncontrollable infections. Written informed consent was obtained from the patients’ caregivers for this study. The experimental protocol received approval from the ethics committees of the hospitals (KY2022-094-02). The clinical characteristics of the patients with DOC are presented in Table 1.

**Table 1:** Clinical data of patients with disorders of consciousness.

No.	Age	Gender	Etiology	Duration (months)	CRS-R	Diagnosis	Threshold intensity (mA)
1	52	Male	TBI	9	10 (232102)	MCS	4.5
2	48	Male	TBI	6	9 (132102)	MCS	4.0
3	59	Male	TBI	4	10 (232102)	MCS	7.0
4	57	Female	ICH	9	13 (144202)	MCS	5.0
5	39	Male	ICH	3	12 (333102)	MCS	3.0
6	39	Male	ICH	3	12 (333102)	MCS	4.0
7	59	Male	TBI	11	17 (444113)	MCS	6.0
8	18	Female	TBI	9	7 (112102)	UWS	3.2
9	28	Male	ICH	5	7 (112201)	UWS	6.0
10	18	Male	TBI	7	7 (112102)	UWS	2.0
11	40	Female	TBI	4	7 (112102)	UWS	3.0

TBI, traumatic brain injury; ICH, intra-cerebral hemorrhage; CRS-R, Coma Recovery Scale-Revised, consists of six subscales: auditory, visual, motor, verbal, communication, and arousal, with higher scores indicating greater awareness; MCS, minimally conscious state; UWS, unresponsive wakefulness syndrome

### 2.2 Study Design

**Fig. 1:**
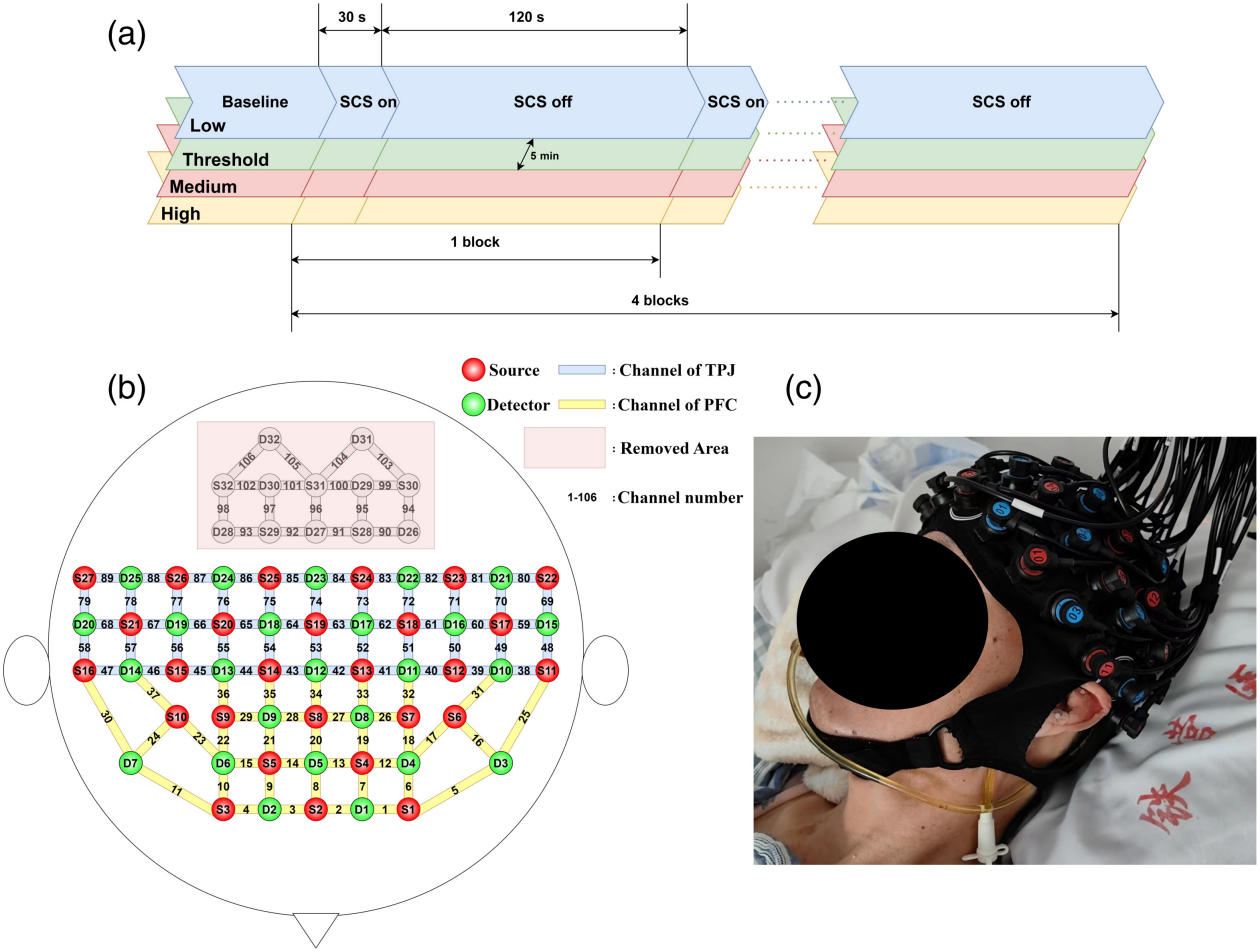
Study design. (a) Experiment paradigm. (b) Arrangement of fNIRS optodes over the brain. (c) Photograph of the experimental setup.

In this study, the experiments were conducted in a quiet and comfortable ward without interruption. The stimulation paradigm was block-designed based on the findings of our previous studies.^12^^,^^13^ The experimental protocol was categorized into four sessions based on the stimulation intensity that reaches the personal motor threshold (defined as the minimal intensity of stimulus capable of producing visible movements in the upper limbs,^7^^,^^18^^,^^19^ the threshold values are shown in Table 1). Specifically, the low-intensity group (50% threshold), also referred to as the sub-threshold group; the threshold-intensity group (100% threshold); the medium-intensity group (125% threshold); and the high-intensity group (150% threshold) yield four conditions. Other stimulation parameters included a frequency of 70 Hz and a pulse width of 210  μs.^10^ Each session consisted of a 60-s baseline followed by four blocks, each block consisting of 30-s stimulation periods followed by 120-s intervals. Each participant underwent all four sessions. The order of different sessions was pseudo-randomized, and a 5-m rest period was allowed to eliminate the influence of the previous session. The experimental details are shown in Fig. 1(a).

### 2.3 Data Acquisition

The hemodynamic data were acquired using the BS-7000S (YIRUIDE Medical Co., Wuhan, China), which utilizes 690- and 830-nm wavelengths of light to distinguish changes in oxyhemoglobin (HbO) and deoxyhemoglobin (HbR) concentrations of the brain. The arrangement of the fNIRS optodes was based on the international EEG 10 to 20 system. A total of 32 light sources and 32 detectors were placed over the whole brain, totally yielding 106 optical channels [Fig. 1(b)]. The distance between the light source and detector pairs was 30 mm, and the sampling rate was 20 Hz. In addition, we excluded the occipital region channels (sources 28 to 32 and detectors 26 to 32, a total of 17 channels, as shown in Fig. 1(b)) due to potential signal distortion issues and the risk of skin damage caused by the patient’s lying posture. The photograph of the setup is shown in Fig. 1(c).

### 2.4 Data Analysis

Data were processed using the MATLAB 2023a platform (MathWorks Inc., Natick, Massachusetts, United States). First, the raw light intensity data were converted to the relative concentration changes of oxygenated (HbO), deoxygenated (HbR), and total (HbT) hemoglobin based on the modified Beer–Lambert law.^35^^,^^36^ As the HbT concentration is proportional to the changes in regional cerebral blood volume,^37^ our analysis focused primarily on HbT concentrations to specify hemodynamic responses. The hemodynamic data were then low-pass filtered at 0.1 Hz and high-pass filtered at 0.01 Hz to remove task-unrelated noise. The correlation-based signal improvement method was used to correct motion artifacts.^38^ Channels exhibiting a high coefficient of variation (CV>25%) were excluded from subsequent analysis, yielding an average exclusion rate of 8.6% across participants (calculated from remaining channels after occipital region removal).

The brain regions were defined as shown in Fig. 1(b), with channels 1 to 37 assigned to the PFC and channels 38 to 89 to the TPJ. The mean values for each region were calculated from the qualified channels and used for further analysis. Each dataset comprised four blocks of 155 s, divided into the pre-SCS phase (−5 to 0 s), the on-SCS phase (0 to 30 s), and the post-SCS phase (30 to 60 s). Quantitatively, the mean and peak values of the HbT concentration during the on-SCS phase (0 to 30 s) and the HbT mean during the pre-SCS phase, as well as the post-SCS phase, are assessed. In addition, the number of activated channels (defined as those with a positive HbT mean during the on-SCS period) and the net area under the curve (AUC) during the ISI phase (30 to 150 s) were extracted at the all-channel level for further quantification. BrainNet Viewer^39^ was used for results visualization.

For statistical analysis, paired sample t-tests and one-way analysis of variance (ANOVA) were performed using the SPSS 27.0 software (IBM Corporation, New York, United States), and the results were visualized by GraphPad Prism 10.0 (GraphPad Software, San Diego, United States). For *post hoc* analysis, the least significant difference test was employed. The results are presented as means ± standard error unless otherwise noted. Differences were considered significant at p<0.05.

## 3 Results

**Fig. 2:**
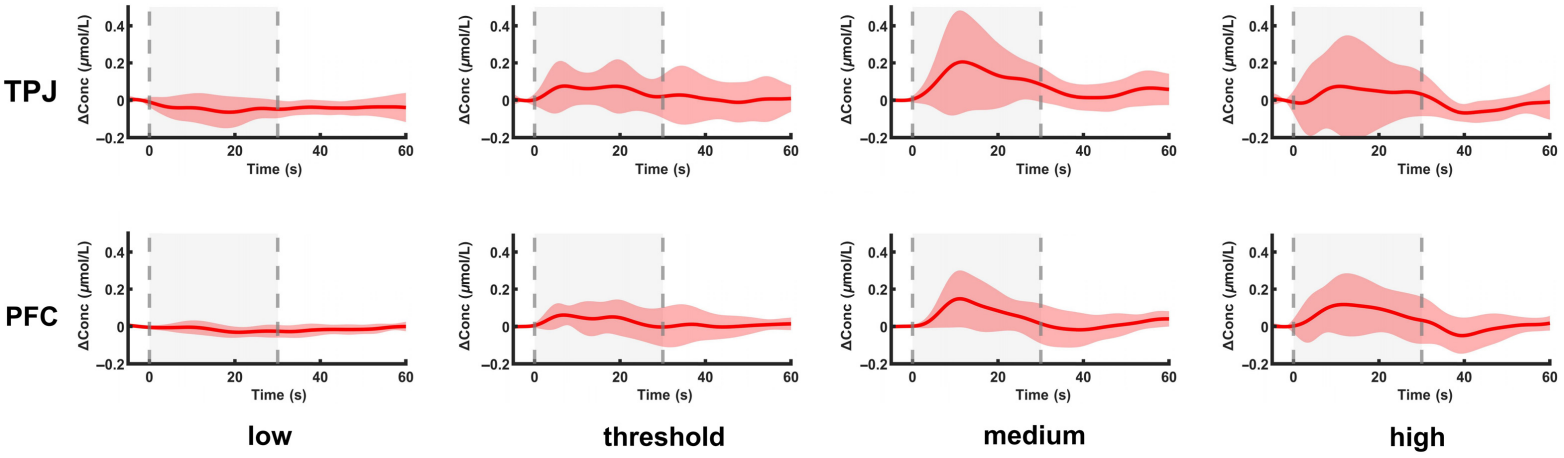
Time course of group-averaged HbT concentration changes in the TPJ and PFC. The red shaded area surrounding each curve represents the standard deviation. The grey shaded region indicates the on-SCS period. TPJ, temporo-parietal junction; PFC, prefrontal cortex; low, low-intensity group; threshold, threshold-intensity group; medium, medium-intensity group; high, high-intensity group.

Group-averaged hemodynamic responses of the DOC patients during the SCS procedure in the PFC and TPJ areas are illustrated in Fig. 2. During the pre-SCS period (−5 to 0 s), HbT concentrations remained stable at baseline levels in both regions of interest (ROIs). After the onset of SCS, the medium-intensity group (medium) exhibited the most pronounced hemodynamic response in both the TPJ and PFC regions, with peak concentrations observed around 15 to 20 s. The high-intensity group (high) showed a similar, albeit slightly lower response, compared with the medium intensity. The threshold-intensity group (threshold) displayed an initial increase, peaking at a lower level and returning to baseline more rapidly. The low-intensity group (low) had minimal changes from baseline, with some decline after the onset of SCS observed. During the post-SCS period (30 to 60 s), all groups except for the low-intensity group showed a decline in hemodynamic response, with the medium intensity group maintaining a higher level above baseline compared with the other groups. The high-intensity and threshold-intensity groups showed moderate levels, whereas the low-intensity group remained close to baseline throughout.

**Fig. 3:**
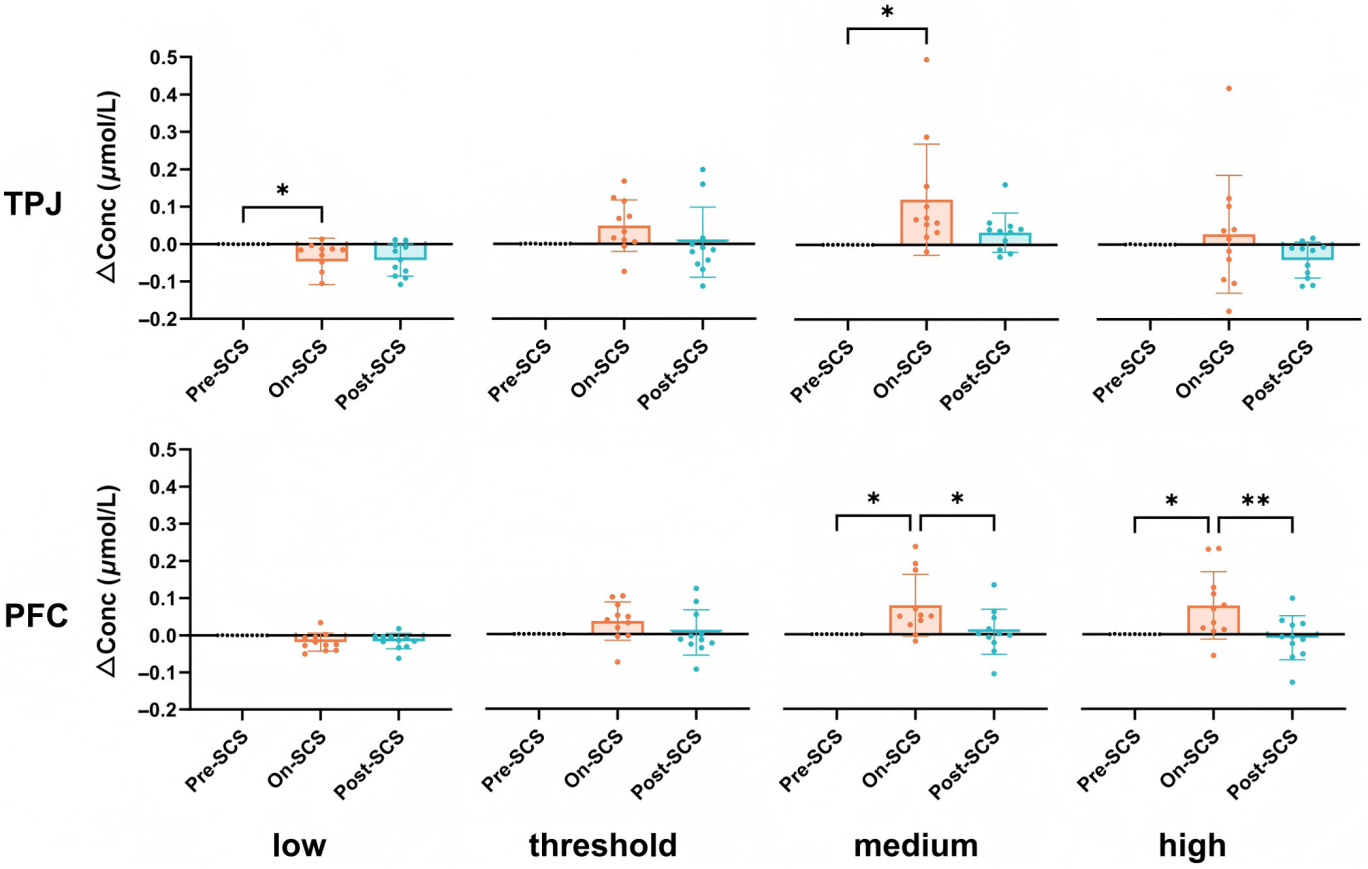
Comparison of HbT concentrations at different stimulation intensities during the pre-, on-, and post-SCS periods. Each point in the figure represents the mean value for an individual patient in pre-SCS phase (−5 to 0 s), the on-SCS phase (0 to 30 s), and the post-SCS phase (30 to 60 s). TPJ, temporo-parietal junction; PFC, prefrontal cortex; SCS, spinal cord stimulation; low, low-intensity group; threshold, threshold-intensity group; medium, medium-intensity group; high, high-intensity group. *P<0.05 and **P<0.01.

To quantify the hemodynamic responses, the mean HbT values in different periods were calculated. The hemodynamic responses among different conditions were compared using one-way ANOVA. The results are shown in Fig. 3. Significant differences were observed among the pre-SCS, on-SCS, and post-SCS groups in the low-intensity group of TPJ (ANOVA: F(2,30)=3.821, P=0.0333). The low-intensity group showed a decline during the on-SCS period compared with the pre-SCS period (*post hoc*: P=0.0483 in the TPJ). The threshold intensity group did not show any significant differences among the three time periods in either brain region. The medium-intensity group exhibited significant changes in HbT concentration during the experimental periods (ANOVA: F(2,30)=5.159, P=0.0119 for TPJ; F(2,30)=5.845, P=0.0072 for PFC). Specifically, this group showed a significant increase in HbT concentration during the on-SCS period compared with baseline (*post hoc*: P=0.0112 in the TPJ and P=0.0119 in the PFC) with no significant differences between the on-SCS and post-SCS periods in the TPJ. However, in the PFC, there was a significant decrease in the post-SCS period compared with the on-SCS period (*post hoc*: P=0.0220). The high-intensity group also demonstrated notable changes. In the PFC, significant differences were observed across the pre-SCS, on-SCS, and post-SCS periods (ANOVA: F(2,30)=6.396, P=0.0049) in the high-intensity group. This group showed a significant increase in HbT concentration during the on-SCS period (*post hoc*: P=0.0185) and a subsequent significant decrease in the post-SCS period (*post hoc*: P=0.0075). No significant differences were observed in the TPJ across the three periods in the high-intensity group.

**Fig. 4:**
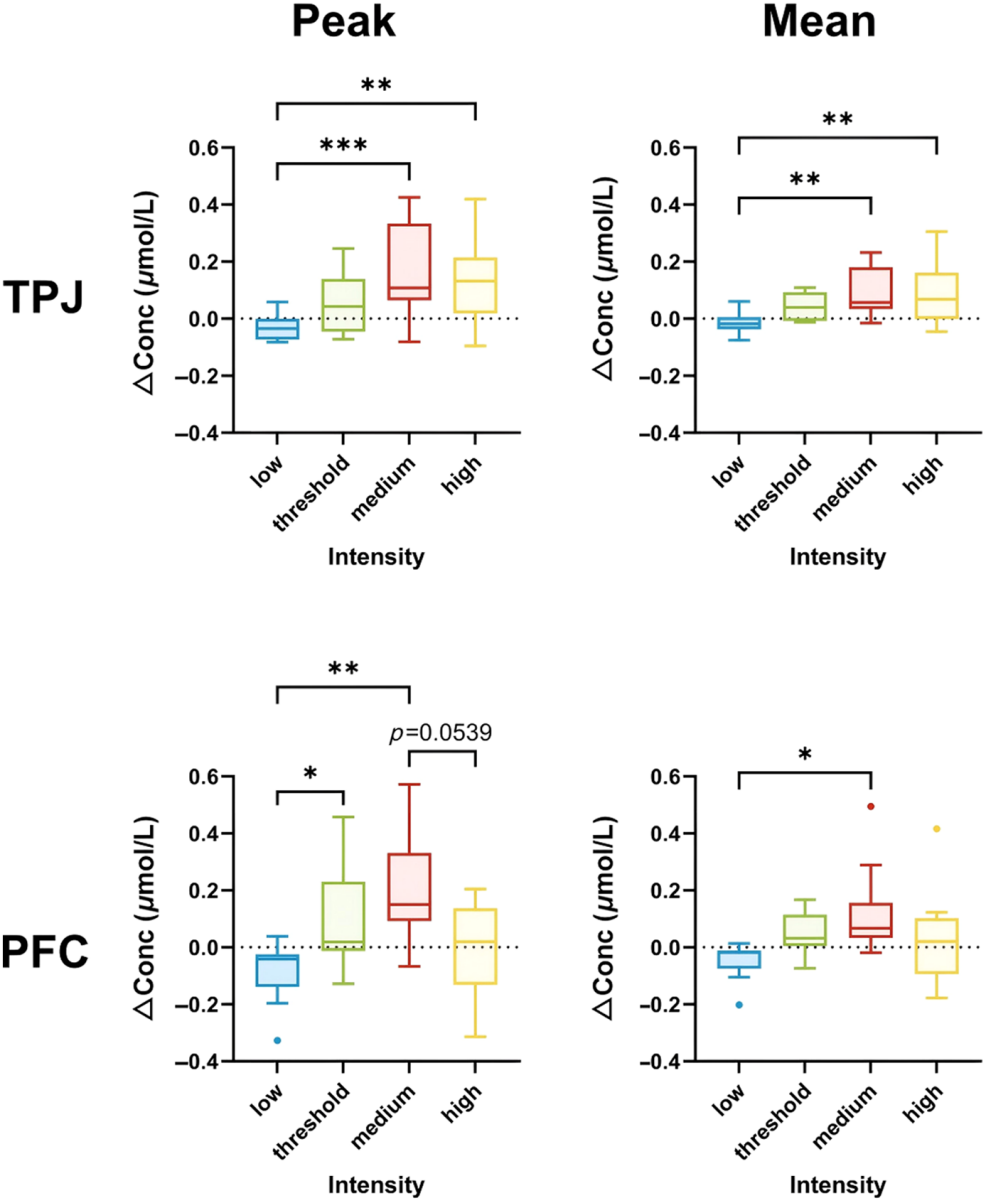
Peak and mean values of the HbT concentrations between two ROIs among the four SCS intensities. Comparison of the changes in the peak and mean of the hemodynamic responses between two ROIs among the four groups. TPJ, temporo-parietal junction; PFC, prefrontal cortex; low, low-intensity group; threshold, threshold-intensity group; medium, medium-intensity group; high, high-intensity group. *P<0.05, **P<0.01, and ***P<0.001.

To further compare the differences among stimulation intensity groups in the two ROIs, the mean and peak HbT concentrations during the on-SCS period were extracted. The results are presented in Fig. 4. In the PFC, significant group differences were observed in both mean and peak HbT concentrations across the four groups (ANOVA: F(3,40)=5.122, P=0.0043 for mean; F(3,40)=5.223, P=0.0039 for peak). The medium-intensity group exhibited a significant increase in both mean and peak HbT concentrations compared with the low-intensity group (*post hoc*: P=0.0082 for mean and P=0.0009 for peak). Similarly, high-intensity SCS led to significant increases in the mean and peak HbT concentrations in the PFC compared with low-intensity SCS (*post hoc*: P=0.0088 for mean and P=0.0033 for peak). The TPJ also exhibited significant group differences in both mean and peak HbT concentrations (ANOVA: F(3,40)=3.700, P=0.0193 for mean; F(3,40)=4.033, P=0.0135 for peak), and significant differences in the mean HbT concentrations were observed only between the medium-intensity and low-intensity groups (*post hoc*: P=0.0104). For peak HbT concentrations in the TPJ, the threshold-intensity group showed a significant increase compared with low-intensity SCS (*post hoc*: P=0.0487), whereas the high-intensity group exhibited a noticeable trend of decrease compared with the medium-intensity group, with this reduction showing a trend toward significance (*post hoc*: P=0.0539).

**Fig. 5:**
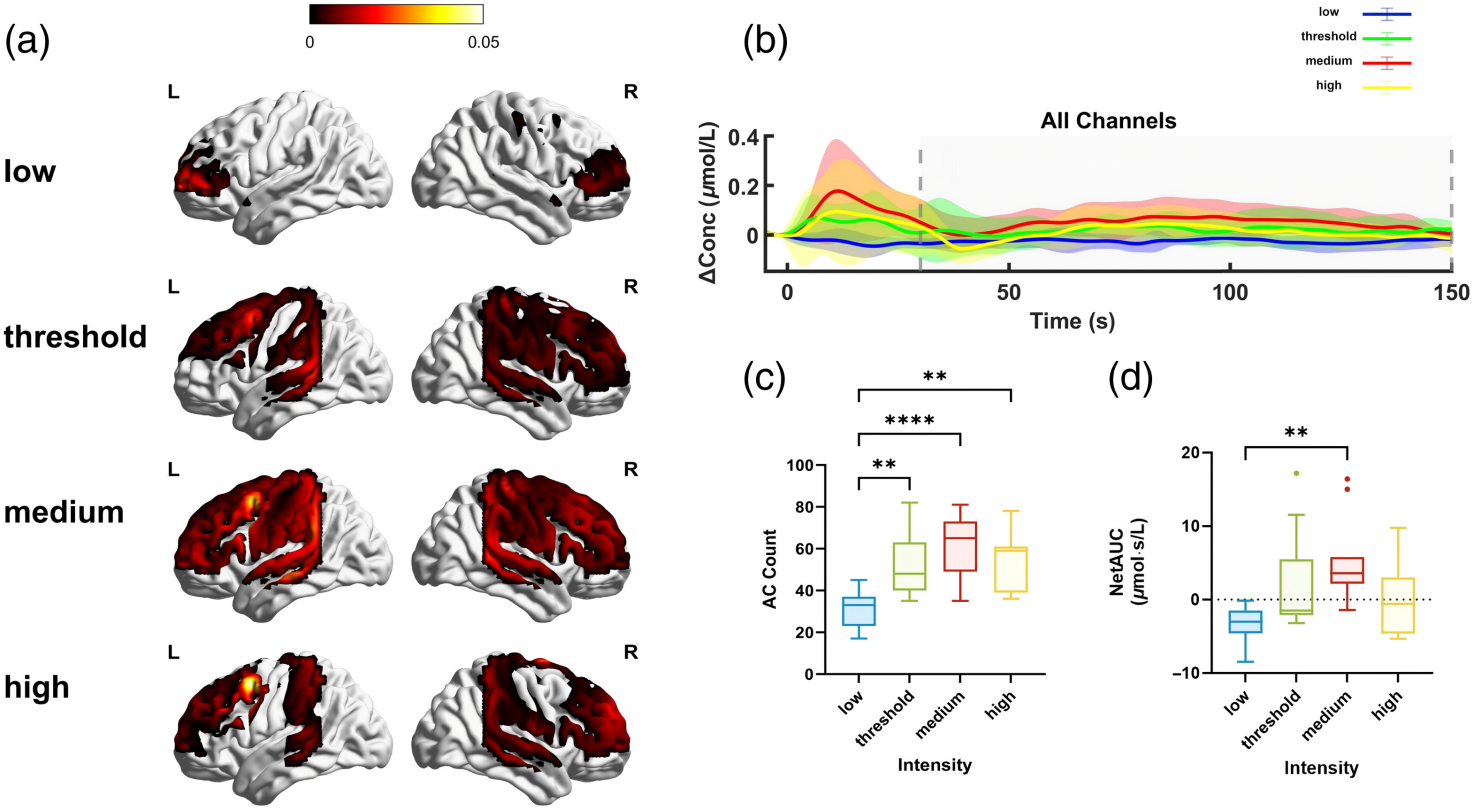
(a) Activation levels in the left and right hemispheres under different stimulation intensities. The surface color represents the mean HbT (positive only) during the on-SCS period, visualized by BrainNet Vierwer.^39^ (b) Group-averaged HbT in each block across all channels (CV>25% excluded). The inter-stimulus interval phase (30 to 150 s) is indicated by the gray interval among the dashed lines. (c) Number of activated channels among different stimulation intensities. (d) Net AUC values across all four stimulation intensities. Error bars indicate the standard errors of the mean. Low, low-intensity group; threshold, threshold-intensity group; medium, medium-intensity group; high, high-intensity group. *P<0.05, **P<0.01, and ****P<0.0001.

To evaluate which stimulation intensity group activates more channels, we assessed the number of activated channels (defined as channels with a positive HbT mean during the on-SCS period) across the four groups and found differences among them(ANOVA: F(3,40)=10.05, P<0.0001). Compared with the low-intensity group, the number of activated channels significantly increased in the threshold-intensity, medium-intensity, and high-intensity groups (*post hoc*: P=0.0019 for threshold, P=0.0001 for medium, and P=0.0030 for high), as shown in Fig. 5(c). However, no significant differences were observed among the supra-threshold intensity groups (ANOVA: F(2,30)=1.047, P=0.3634). Also, we explored the net AUC during the ISI period to assess the sustained HbT increase due to SCS across different intensity groups [Fig. 5(b)]. Although ANOVA revealed significant overall differences among the four groups (ANOVA: F(3,40)=5.665, P=0.0025), *post hoc* analysis showed that this effect was specifically driven by a significant difference between the medium-intensity and low-intensity groups (*post hoc*: P=0.0055), as illustrated in Fig. 5(d).

## 4 Discussion and Conclusion

### 4.1 Threshold Effect and Inverted U-Shaped Dose–Response Curve

All four intensity groups maintained stable HbT levels during the baseline period, indicating the absence of significant external stimuli and the synchronization of SCS onset with fNIRS recording. After SCS initiation, the HbT concentrations in three supra-threshold groups (threshold, medium, and high groups) increased during stimulation and gradually returned to baseline. The observed dynamic patterns aligned with established responses: they matched cognitive task-evoked hemodynamics in healthy subjects^40^ and paralleled SCS-induced hemodynamic changes in DOC patients.^12^ Such responses are believed to play a positive role in the recovery of consciousness levels in DOC patients. In our study, all supra-threshold groups exhibited positive and meaningful HbT concentration changes. When comparing the number of activated channels across different conditions, all three supra-threshold groups showed significant improvements compared with the sub-threshold group, with no significant differences among the supra-threshold groups themselves. Sheth et al.^41^ discovered a similar threshold effect on a rodent model where neural activity of insufficient intensity fails to provoke a corresponding blood flow response. The current findings further confirmed the existence of a threshold effect, which is that when the stimulation intensity reaches above the motor threshold, the blood flow also reaches a threshold that rises with the intensity.

Notably, a significant decrease in HbT concentration was observed during the on-SCS period in the sub-threshold group during the on-SCS period in TPJ. Figure 5(a) also showed that in the low-intensity group, only a small portion of PFC is activated during the on-SCS phase, suggesting a negative hemodynamic response in most regions compared with the baseline. It has been found that compared with 90% motor threshold, SCS at 60% of motor threshold significantly inhibits neural activity in the anterior cingulate cortex evaluated by EEG.^42^ Such inhibitory effects are more desirable for sedation but may not be preferred for awakening DOC patients due to the negative hemodynamic response SCS induced.

Supra-threshold stimulation can effectively activate cortical blood flow responses. However, the activation effect does not increase linearly with intensity; instead, it follows an inverted U-shaped dose–response curve. The medium-intensity group showed more effective activation during and after stimulation than the other supra-threshold groups. Although the high-intensity group showed improvements in most indicators compared with the low-intensity group, it exhibited a decrease in peak values in the TPJ region compared with the medium-intensity group. Figure 5(a) also indicates that in the supra-threshold groups, most regions are activated. The most balanced activation across the brain is observed in the medium-intensity group, whereas the other groups still show some areas being deactivated. This further confirms the existence of the inverted U-shaped dose–response curve. Similar inverted U-shaped dose–response curves have been observed in other experiments. For instance, Chung et al.^14^ found that stimulation intensity of repetitive TMS set at 75% of the motor threshold intensity induces greater neurophysiological changes compared with 50% and 100% intensities. Mosayebi-Samani et al.^16^ demonstrated that cathodal tDCS showed non-linear effects in the motor cortex, with 1 mA inducing inhibition, 2 mA producing excitation, and 3 mA again resulting in inhibition. Analogously, in SCS, the dose–response relationship between stimulation intensity and hemodynamic response is also non-linear, with peak efficacy observed at a medium intensity, and the inflection point at which the hemodynamic response starts to attenuate is between medium and high intensities.

Taken together, these findings provide evidence that optimal cortical activation in DOC patients is achieved not by simply increasing stimulation intensity but by carefully titrating to a medium level above the motor threshold. This is because of the threshold effect we found, which demonstrates that intensities below the motor threshold fail to elicit sufficient cortical activation, and the inverted U-shaped dose–response relationship observed, which shows that excessive intensity reduces effectiveness.

### 4.2 Hemodynamic Response During Inter-Stimulus Interval

To date, treating DOC with SCS has been using intermittent dosing, in which phases of on-SCS are alternated with phases of ISI.^7^^,^^10^^,^^12^ This is because excessive and continuous stimulation may lead to neuronal fatigue and damage and could worsen motor function.^13^^,^^43^ Our findings provide empirical support for this clinical practice by showing distinct post-stimulation hemodynamic patterns across intensities. In our study, both the medium- and high-intensity groups showed a significantly decreased HbT toward the baseline after initial stimulation and remained around the baseline for ∼30  s after SCS turned off, defining the post-SCS period. In the high-intensity group, the mean HBT levels in the TPJ during the post-SCS phase are overall lower than baseline levels (P<0.05), which means an inhibitory effect on cortical neural activity after excessive stimulation. In contrast, the medium-intensity group shows an above-baseline HbT level in the TPJ region during the post-SCS phase (P<0.05), indicating a prolonged activation effective while avoiding potential inhibition.

Extending the post-SCS time scale to the ISI period (120-s post-stimulation), we observed that the dynamic HbT changes deviated further from the baseline. After the initial increase due to SCS, HbT levels gradually decreased, then gradually increased, remaining above baseline throughout the ISI period, aligning with our previous studies.^12^ Only the medium-intensity group showed significant increases in HbT levels during the ISI period compared with the low-intensity group. This indicates medium intensity maintains a beneficial after-effect, which may help sustain neural excitability and recovery-related processes. Our previous research established that sustained high HbT levels during the ISI period correlate with favorable prognosis in DOC patients,^13^ suggesting medium-intensity stimulation may yield better clinical outcomes.

### 4.3 Mechanistic Insights of Individual and Regional Variability

Upper limb twitches are commonly regarded as indicators of effective stimulation, indicating activation of spinal motor neurons, which may play a crucial role in facilitating the neurorehabilitation of motor function.^11^ The motor threshold can vary significantly among different individuals due to several clinical factors, including pathological conditions, electrode placement, and upper limb motor function.^18^ Using the motor threshold as 100% intensity and delineating different levels of stimulation based on this threshold is considered a reasonable approach. Our results support this method by showing that, despite differences in absolute intensities across patients, comparable hemodynamic response patterns were observed when stimulation was calibrated relative to each patient’s motor threshold. Although some studies have employed absolute intensity settings,^9^^,^^19^ the use of motor threshold as a reference in clinical practice is more common and has been demonstrated effective in previous research.^7^^,^^12^^,^^44^^,^^45^

In studies using SCS for other populations, the therapeutic window can be defined based on patient feedback. For instance, a research established the lower bound as the evoked compound action potentials (ECAP) amplitude at the perception threshold and the upper bound as the ECAP amplitude at the maximum tolerance threshold.^46^ However, for patients with DOC, explicit feedback required for closed-loop adjustment is unavailable. Although the widely accepted motor threshold serves as the lower bound for SCS therapy in DOC, the upper limit of the therapeutic window has never been established. Using fNIRS technology, we propose defining the upper bound as the stimulation intensity at which hemodynamic responses begin to decline. Our findings demonstrate distinct regional variations in therapeutic upper limits: the PFC exhibited broader intensity tolerance with peak responses spanning 125% to 150%, reflected by similar hemodynamic patterns at medium and high intensities, whereas the TPJ demonstrated a constrained therapeutic window with optimal response near 125% intensity.

In a research on deep brain stimulation, the therapeutic window is described as dependent on parameters, stimulation target, and other factors,^47^ suggesting that in our research, under the same parameters, the therapeutic window differs due to the variability of the two regions. It can be inferred that the TPJ, being more directly involved in sensory processing and integration, may be more sensitive to the intensity of SCS. In contrast, the PFC’s role in higher-order cognitive functions may require more robust activation to manifest significant hemodynamic changes.

In addition, previous research using TMS reported consistent differences in the natural frequencies between the frontal and parietal lobes across individuals,^48^ indicating inherent variations in their response properties. These findings suggest that different brain regions may exhibit unique optimal therapeutic windows in response to SCS due to their distinct neurophysiological characteristics, thereby accounting for the observed variation in the dose–response curves. According to a previous study, the TPJ has been shown to exhibit significantly increased neural fiber density in a patient transitioning from UWS to MCS, underscoring the central role that the TPJ plays in the process of consciousness recovery.^49^ However, over-stimulating the TPJ poses a risk of damaging its intricate neural fiber and impeding the recovery process. Consequently, medium-intensity stimulation appears to be the most effective approach, as it provides robust activation of the PFC while avoiding potential damage to the neural fibers of the TPJ. This regional specificity in response to SCS should guide the development of targeted neuromodulation strategies, thereby enhancing the overall efficacy of SCS and other neuromodulation in restoring consciousness functions in DOC patients.

### 4.4 Limitations and Future Directions

Despite the promising findings, several limitations must be acknowledged. The study’s relatively small sample size, combined with potential variations in SCS-induced activation patterns across different etiologies (notably in TBI), necessitates larger-scale investigations to confirm these results and generalize them to broader DOC patient populations. What is more, this study focused exclusively on hemodynamic effects of SCS intensity in DOC patients. Future studies should incorporate both short-term physiological monitoring and longitudinal assessments of behavioral recovery and consciousness-level changes to provide deeper insights into SCS therapeutic effects. Moreover, although our team has explored the effects of different stimulation intensities, frequencies, and ISIs of SCS on DOC patients, the intrinsic mechanisms underlying these parameter-dependent hemodynamic responses remain incompletely elucidated. In further studies, different combinations of parameters (e.g., intensity and frequency, pulse width, and waveform) and different combinations of neuroimaging techniques (e.g., EEG or fMRI) can provide a more comprehensive understanding of the mechanisms of SCS.

## Data Availability

The data that support the findings of this article are not publicly available due to privacy and ethical concerns. They can be requested from the corresponding authors on reasonable request.
